# Novel *Trichoderma polysporum* Strain for the Biocontrol of *Pseudogymnoascus destructans*, the Fungal Etiologic Agent of Bat White Nose Syndrome

**DOI:** 10.1371/journal.pone.0141316

**Published:** 2015-10-28

**Authors:** Tao Zhang, Vishnu Chaturvedi, Sudha Chaturvedi

**Affiliations:** 1 Mycology Laboratory, Wadsworth Center, New York State Department of Health, Albany, New York, United States of America; 2 Department of Biomedical Sciences, School of Public Health, University at Albany, Albany, New York, United States of America; Università degli Studi di Napoli Federico II, ITALY

## Abstract

White-nose syndrome (WNS), an emerging disease of hibernating bats, has rapidly spread across eastern North America killing millions of bats. *Pseudogymnoascus destructans* (*Pd*), the sole etiologic agent of WNS, is widespread and persistent in bat hibernacula. Control of *Pd* in the affected sites is urgently needed to break the transmission cycle while minimizing any adverse impact on the native organisms. We isolated a novel strain of *Trichoderma polysporum* (*Tp*) from one of the caves at the epicenter of WNS zoonotic. Detailed experimental studies revealed: (1) *Tp* WPM 39143 was highly adapted to grow at temperatures simulating the cave environment (6°C-15°C), (2) *Tp* WPM 39143 restricted *Pd* colony growth in dual culture challenges, (3) *Tp* WPM 39143 caused four logs reduction of *Pd* colony forming units and genome copies in autoclaved soil samples from one of the WNS affected caves, (4) *Tp* WPM 39143 extract showed specific fungicidal activity against *Pd* in disk diffusion assay, but not against closely related fungus *P*. *pannorum* (*Pp*), (5) *Tp* WPM 39143 extract retained inhibitory activity after exposure to high temperatures, light and proteinase K, and (6) Inhibitory metabolites in *Tp* WPM 39143 extract comprised of water-soluble, high polarity compounds. These results suggest that *Tp* WPM 39143 is a promising candidate for further evaluation as a biocontrol agent of *Pd* in WNS affected sites.

## Introduction

White-nose Syndrome (WNS), caused by *Pseudogymnoascus destructans* (*Pd)*, has devastated bat populations across eastern North America for almost a decade [1–7]. A number of bat species are affected with the most vulnerable being little brown bats (*Myotis lucifugus*), Indiana bats (*M*. *sodalis*), northern long-eared bats (*M*. *septentrionalis*), and tricolored bats (*Perimyotis subflavus*) [5, 8]. *Pd* invades the skin of hibernating bats including muzzles, ears, and wings. The affected animals are noticeably emaciated, and many fail to survive hibernation [9–11].

The underlying mechanisms of *Pd* infectivity and its precise ecological niche are largely unknown. Historically, *Pd* appears to be well established in European caves, and European bats harboring the fungus exhibit pathology similar to that seen in North American bats, with no recorded mass mortality [12–14]. To date, all investigations of *Pd* indicate that it is well adapted to cold conditions and has a clonal population in the US [15, 16]. Several DNA and culture based studies have revealed wide distribution and persistence of *Pd* in sediment and swab samples from bat hibernacula [12, 17–19]. Other published cave fungal surveys indicate that *Pd* could survive and persist in bat hibernacula for prolonged periods, and this has a dramatic impact on both disease management and epidemiology [19]. Recently, a mycobiome study found a diversity of fungi inhabiting WNS caves and mines and signs of local adaptation by *Pd* [20]. Further studies are needed to understand the pathogenesis of WNS and to develop control strategies that include alleviation or remediation of *Pd* to break the transmission cycle, especially by reducing the fungal burden available for new infections. Recently, some bacteria have been proposed as biocontrol agents, including naturally occurring bacteria [21] or volatiles produced by bacteria [22, 23].

There is a vast volume of literature on the biocontrol potential of *Trichoderma* spp. and several highly effective preparations incorporating these fungi are now approved for commercial use for the biocontrol of agriculture pests in the US and other parts of the world [24, 25]. The most important biocontrol agents against plant pathogenic fungi are *Trichoderma virens* von Arx, Beih [26, 27], *T*. *harzianum* Rifai [28, 29], *T*. *viride* Pers:Fr [30], and *T*. *atroviride* sc1 [31]. Optimum growth temperatures differ among *Trichoderma* spp. and previous studies have largely focused on mesophilic groups (22°C~35°C) [24, 32–34]. It has been found that *Trichoderma* spp. did not protect germinating seeds from soil-borne diseases caused by cold-tolerant phytopathogenic fungi during cold and wet autumn and spring seasons [35, 36]. To date, there is relatively little information available about psychrotolerant *Trichoderma* spp. and their biocontrol potentials. *Trichoderma polysporum* was shown to have biocontrol activity outside of caves against a variety of other fungi including *Armilaria gallica*, *Fomus annosus*, and *Ceratocystis paradoxa* [37–39].

The present study describes a psychrotolerant strain of *Trichoderma polysporum* (*Tp*) WPM 39143, which was isolated from a cave at the epicenter of the WNS zoonotic. Since *T*. *polysporum* WPM 39143 grew relatively well at low temperatures common in the cave environment [40], the objective of this investigation was to determine if it exerted inhibitory activity against *Pd* in laboratory media and soil matrices.

## Materials and Methods

### Fungal strains and media

*Trichoderma polysporum* (*Tp*), recently isolated from air sample 39143 from William Preserve Mine (WPM), NY [20], *Trichoderma harzianum* (*Th*) Rifai strain T22 purchased from BioWorks, Inc. Victor, NY, USA, *T*. *atroviride* (*Ta*) obtained from ARS fungal collection, Cornell University, NY, USA, *Pseudogymnoascus destructans* (*Pd*) M1379 and *Pseudogymnoascus pannorum* (*Pp*) M1372 [41] were part of this investigation. All fungi were maintained on potato dextrose agar (PDA, Difco) slants at 15°C. Fungal cultures were also stored in 15% glycerol at -70°C. Other media used were Sabauraud dextrose agar (SDA) with or without cycloheximide (400 μg/ml), rice fermentation medium (100 g rice in 150 ml of distilled water) [42], and yeast extract peptone dextrose (YPD) agar. All the experiments were performed in the biosafety cabinet 2.

### Phylogenetic analysis of *Tp* WPM 39143

Genomic DNA from *Tp* WPM 39143 was extracted using thermolysis phenol extraction protocol as described in a published study [43]. In brief, approximately 5x5 mm of fungal culture was removed from the agar surface, placed in 300 μl of modified genomic DNA extraction buffer (100 mM Tris [pH 8.0], 10 mM EDTA, 2% SDS, 1.4 M NaCl, 1% CTAB, 0.4 μg/ml proteinase K), and the mixture incubated at 65°C for 1 h followed by chloroform:isoamyl alcohol (24:1) extraction, precipitation with isopropanol and washing with 70% ethanol. The precipitated DNA was centrifuged at 12,000 RPM and the resulting pellet was dried and finally dissolved in 50 μl of Tris-EDTA (TE) buffer. The extracted DNA was amplified for the internal transcribed spacer (ITS) and D1/D2 regions of the ribosomal gene with primer set ITS1-ITS4 and NL1-NL4, respectively [44]. The PCR was performed with proof reading KlenTaq DNA polymerase (Sigma-Aldrich, St. Louis, MO, USA) with initial denaturation at 95°C for 3 min, followed by 30 cycles of denaturation at 94°C for 1 min, annealing at 55°C for 1 min, and extension at 68°C for 2 min and the final extension at 68°C for 10 min. The PCR amplicons were sequenced, assembled, and edited using Sequencher 4.6 software (Gene Codes Corp., Ann Arbor, MI, USA) and BLAST searched against two databases: GenBank (www.ncbi.nlm.nih.gov/) and CBS-KNAW (www.cbs.knaw.nl/).

Multiple alignments of ITS and D1/D2 sequences of *Tp* WPM 39143 and selected GenBank sequences were done using the CLUSTALX 1.81 [45] and MAFFT programs [46]. A phylogenetic analysis of the aligned sequences was done using the neighbor-joining (NJ) method with 1,000 bootstrap replicates using MEGA 5.1 [47]. The Dictionary of the Fungi and UniProt (http://www.uniprot.org/taxonomy/) served as the source of taxonomic references for fungal species [48].

### Growth optima of *Tp* WPM 39143

In order to assess the biocontrol potential, we first compared the growth temperature range of *Tp* WPM 39143 with that of two well -characterized biocontrol fungi *Th* and *Ta*. These strains were point inoculated on the center of PDA and YPD agar plates, and cultures were incubated at 6, 10, 15, and 22°C. The diameter of the fungal colony was measured at 9 and 20 days post-incubation.

### Dual culture challenge studies of *Pd* and *Tp* WPM 39143

The dual challenge experiments were performed on the laboratory medium (PDA) and soil matrices. Approximately 15 μl of *Pd* conidial suspension (10^7^ conidia/ml) was inoculated close to the edge on one side of a PDA agar plate. Following incubation for 10 days at 15°C, approximately 15 μl of conidial suspension (10^5^ cells/ml) of *Tp* WPM 39143 was placed on the opposite side of the plates. Similarly, a conidial suspension of *Th* (10^5^ cells/ml) was also placed on the opposite side of the *Pd* culture plate in a separate experiment. The interactions between *Pd* and *Tp* or *Th* were assessed at 2 and 4 weeks post-inoculation at 15°C. The *Pd* culture alone served as a control.

For the dual challenge experiment simulating a natural setting, one soil sample (dark black in color with fine granular consistency) collected from Aeolus Cave on September 18, 2013, was used. In brief, the soil sample was weighed, and 3 grams placed into sterile 10-ml screw capped Econo Glass Vials^TM^ (PerkinElmer, Waltham, MA, USA). Vials were autoclaved at 15 psi for 30 min. The pH of the autoclaved samples was determined by pulverizing the soil sample with 10 ml of sterilized distilled water and placing a drop of this suspension on to pH indicator strips ranging from pH 0–6 and pH 5–10. A loopful of autoclaved soil sample was streaked onto YPD agar plates, and the plates were incubated at 15°C for one week to confirm sterility.

Three vials, each containing 3 g of soil sample, were inoculated with 100 μl of *Pd* conidial suspension (3×10^5^ conidia/ml) to get final conidia counts of 1 x 10^4^ conidia/g of soil. The contents of the vials were thoroughly mixed with a sterile spatula, and these vials were incubated for one week at 15°C. Following incubation, each *Pd*-containing vial was seeded with either 100 μl of *Tp* WPM 39143 or *Th* conidial suspension (3×10^5^ conidia/ml) to get final conidia counts of 1 x 10^4^ conidia/g of soil. One *Pd*-containing vial was also inoculated with 100 μl of sterile distilled water, which served as a control. All the vials were incubated at 15°C for an additional 35 days.

### Recovery of *Pd* from dual culture challenge

Following incubation, approximately 1 g of soil sample was removed from each vial, transferred to an autoclaved mortar and pestle and mixed gently to get a homogenous mixture. The samples were weighed, and approximately 100 mg aliquots of homogenous soil sample were transferred into six 2-ml screw cap vials. Three vials each were processed for *Pd* recovery by culture-dependent (CD) and culture-independent (CI) methods as shown in the flowchart (S1 Fig).

For the CD method, the content of each vial was mixed with 500 μl of sterile distilled water, vortexed vigorously for 2 minutes and the tubes were left in an up-right position for 5 minutes to allow settling of soil particles. Approximately, 200 μl of supernatant from each vial was removed and transferred to a new sterile vial. Ten-fold dilutions of the supernatant were prepared, and 100 μl of each dilution was inoculated on to SDA plates with or without cycloheximide in triplicates for the recovery of *Pd*, *Tp* WPM 39143, and *Th*. The culture medium selection was based upon our preliminary experiments wherein *Pd* was found to be resistant to cycloheximide while *Tp* WPM 39143 and *Th* showed 100% growth inhibition (details not shown). All the plates were incubated at 15°C for 7 to 30 days. The *Pd* colonies were counted, and results were expressed as log colony-forming unit (CFU) per gram of soil. The percent *Pd* growth inhibition by *Tp* WPM 39143 or *Th* was calculated as: 1-(*Pd* CFU experiment/*Pd* CFU control) x 100.

For the CI method, the remaining three vials, each containing 100 mg of soil sample with *Pd* alone or *Pd* with *Tp* WPM 39143 or *Th*, were subjected to genomic DNA (gDNA) extraction using SoilMaster^TM^ DNA Extraction Kit (Epicentre, Chicago, IL, USA). The autoclaved pre-treated soil sample was also processed for DNA extraction to assess if it contained any *Pd* gDNA. The extracted DNA from all sample types was suspended in 30 μl of Tris-EDTA (TE) buffer and *Pd* gDNA in these samples were quantitated using real-time PCR assays targeting alpha-L-rhamnosidase gene (ALR) and intergenic spacer region (IGS) of the rRNA gene complex in an IQ5 real-time PCR detection system (Bio-Rad, Hercules, CA), as described previously [49, 50]. In brief, each reaction mixture contained 1× LightCycler Fast Start DNA master hybridization probe mix (Roche Applied Science, Indianapolis, IN, USA), 4 mM MgCl_2_, 1 μM concentration of each primer, 0.25 μM concentration of each probe, and 2 μl of soil DNA in a final volume of 20 μl. The soil sample was also checked for PCR inhibitors by spiking with 1 ng of *Pd* gDNA. The PCR cycling conditions were 10 min at 95°C followed by 15 s at 95°C and 60 s at 60°C for 40 cycles. Each sample was tested in triplicate, and the results were averaged to obtain the cycle threshold (*Ct*)—i.e., the point at which sample fluorescence rises above the background level. A *Ct* value of >40 was considered negative while a *Ct* value of ≤40 was considered positive. Each sample was also run with a non-DNA template (NTC) as a negative control. The dilution series of gDNA from a pure culture of *Pd* (M1379) was used for the generation of standard curve. The average *Ct* counts equivalent to DNA quantity was extrapolated from the standard curve, and then *Pd* genome copies was calculated using formula:

(copies = DNA quantity (ng)x(6.022x10^23^)/genome length (bp)x(1x10^9^)x650 in which the 6.022x10^23^ molecules/mole is Avogadro's number, average weight of a base pair (bp) is 650 Daltons and *Pd* genome length is 30.49 Mb. More details of the formula used are available online (http://cels.uri.edu/gsc/cndna.html). The *Pd* genome copies obtained was calculated per gram of soil. Since IGS rRNA gene complex in fungi is a multi-copy gene [51, 52] and IGS PCR was 100 fold more sensitive than the single copy ALR PCR [50], the IGS was arbitrarily calculated as 100 copies per *Pd* genome. The autoclaved pre-treated soil sample was weakly positive for *Pd* gDNA (mean *Ct* = 38.58) by IGS real-time PCR assay.

### Extraction of crude extracts from *Tp* WPM 39143

*Tp* WPM 39143 and the well-known biocontrol fungi, *Th* and *Ta*, were grown on PDA agar at 15°C for 30 days. Fungal growth was gently scraped with a plastic loop, and a spore/hyphal suspension was harvested in sterile distilled water. Approximately 5 ml of spore/hyphal suspension (OD_600_ = 1.0) was seeded into six 500-ml flasks containing 100 g of autoclaved rice fermentation medium, and cultures were then incubated at 22°C for 30 days. Following incubation, the fermented rice substrate was extracted with methanol (3 x 400 ml) by 4 h incubation at 22°C. The methanol-fungal slurry was centrifuged at 8,000 RPM for 10 min. The fungal pellet was removed, and the supernatant evaporated under vacuum. The dry pellet (approximately 7~8 g) was suspended into 30 ml of methanol, which served as a crude extract.

A portion of the crude extract was assessed for stability by exposure to high temperature (45°C), regular light and 10 μl of proteinase K (37° and 45°C) for a total of 20 h each.

### HPLC fractionation of *Tp* WPM 39143 crude extract

The *Tp* WPM 39143 crude extract was analyzed for the presence of different metabolites by high-performance liquid chromatography (HPLC). In brief, 20 μl of crude extract from *Tp* WPM 39143 was placed into HPLC column (Agilent Zorbax SB-C18 column, 5 μm, 4.6×250 mm, 1 ml min^-1^) and different fractions were eluted using gradients starting with 5% CH_3_OH in H_2_O to 100% CH_3_OH for 55 min followed by 100% CH_3_OH for 5 min (12 fractions). The crude extract in the column was further eluted using gradients starting with 100% CH_3_OH to 90% acetonitrile in H_2_O for 10 min (2 fractions) and final washing with 5% CH_3_OH in H_2_O for 5 min (1 fraction). A total of 15 fractions were collected. This experiment was repeated 10 times and fractions from each run were pooled, concentrated 100 fold, and then each fraction was assessed for anti-*Pd* activity using antimicrobial disc diffusion bioassay.

### Antimicrobial disc diffusion bioassay

The antimicrobial disc diffusion bioassay was performed as described in a previous publication [53]. In brief, conidial suspensions of *Pd* and *Pp* were prepared by gently scraping fungal growth from the agar surface and then passing through a 27-G needle. Conidia were counted microscopically using a hemocytometer, and approximately 100 μl of conidial suspension (10^8^ conidia/ml) was spread on YPD agar plates. The 6 mm autoclaved filter discs (Whatman no. 1) were carefully placed on seeded plates and 15 μl of untreated or treated crude extract or extract purified through HPLC column was placed onto filer discs. Plates were incubated for 5–7 days at 15°C, and the zone of fungal growth inhibition around the disc was measured.

### Statistical Analysis

Data in figures are presented as mean ± SD. All statistical analyses were performed using GraphPad Prism software (GraphPad, San Diego, CA, USA). The comparison of mean value of multiple groups was performed using a two-way ANOVA and comparison of two groups was performed using a two-tailed unpaired t-test. Values were accepted as significant if p ≤ 0.05.

### Nucleotide Sequence Accession Numbers

All nucleotide sequences of *Tp* WPM 39143 (*Hypocrea pachybasioide*) were deposited in GenBank under accession numbers: ITS sequence, KJ494575; 28S rRNA gene partial sequence, KJ494576.

## Results

### Phylogenetic analysis of *Tp* WPM 39143

**Fig 1:**
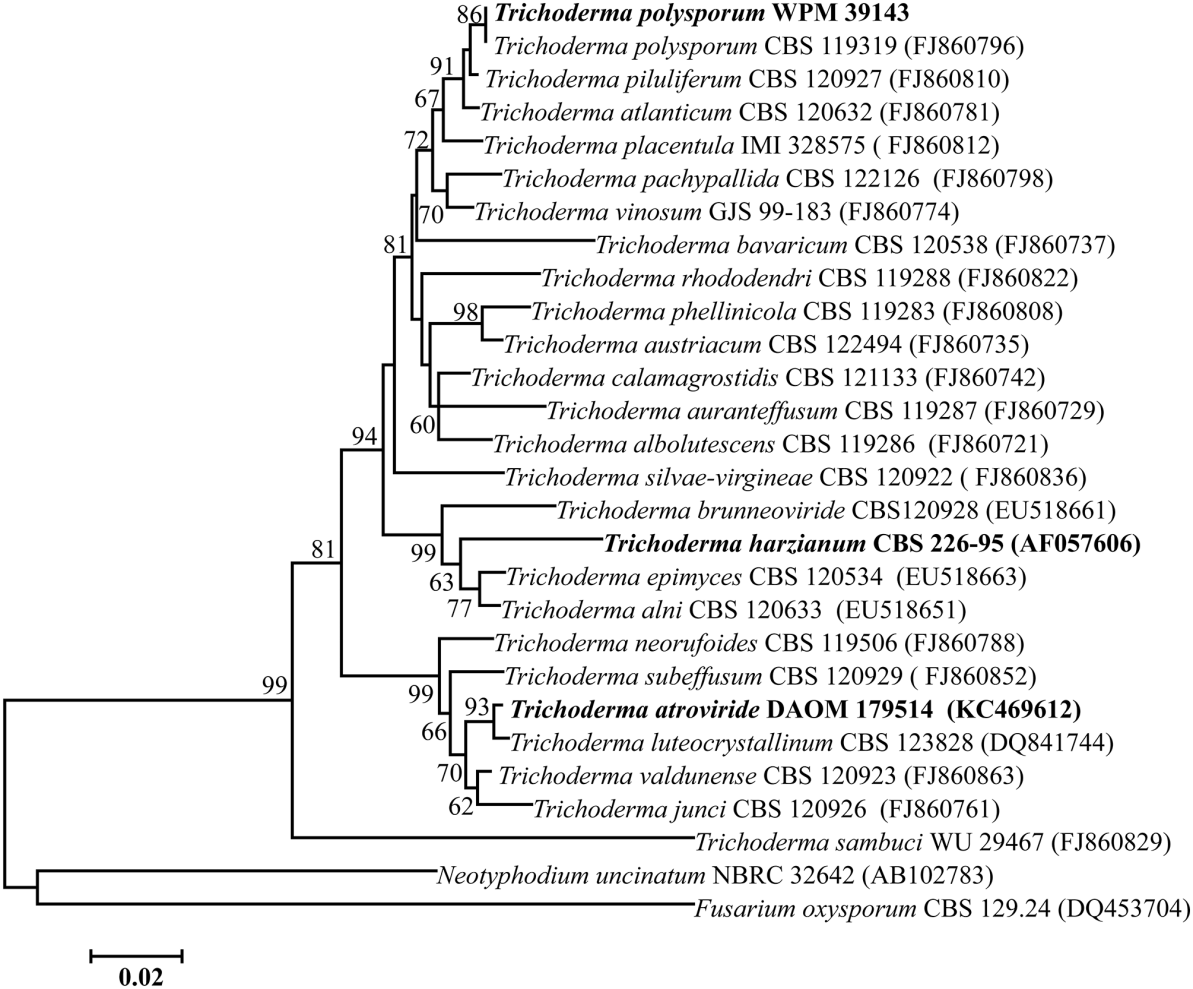
Phylogenetic analysis of *Tp* WPM 39143. The ITS nucleotide sequence of *Tp* WPM 39143 was aligned with similar sequences from 27 taxa of *Trichoderma/Hypocrea* species available in the GenBank. The neighbor-joining method was used to construct the phylogenetic tree. The bootstrap scores are based on 1,000 reiterations. *Fusarium oxysporum* CBS 129.24 and *Neotyphodium uncinatum* NBRC 32642 were used as outgroup.

BLAST search of the ITS sequences of the ribosomal gene of *Tp* WPM 39143 strain showed close homology with *T*. *polysporum* strain CBS 119319 (accession no. FJ860796). Further, phylogenetic analyses using a Neighbor-Joining tree revealed that *Tp* strain WPM 39143 was closely related to *Hypocrea pachybasioide* CBS 119319 (teleomorph of *Trichoderma polysporum*) followed by *T*. *piluliferum*. In contrast, *Tp* WPM 39143 was distantly related to the well-known biocontrol fungi *Th* and *Ta* (Fig 1). Phylogenetic analysis of the D1/D2 region of the 28S ribosomal gene revealed a similar placement of respective genera (data not shown).

### Growth characteristics of *Tp* WPM 39143

**Fig 2:**
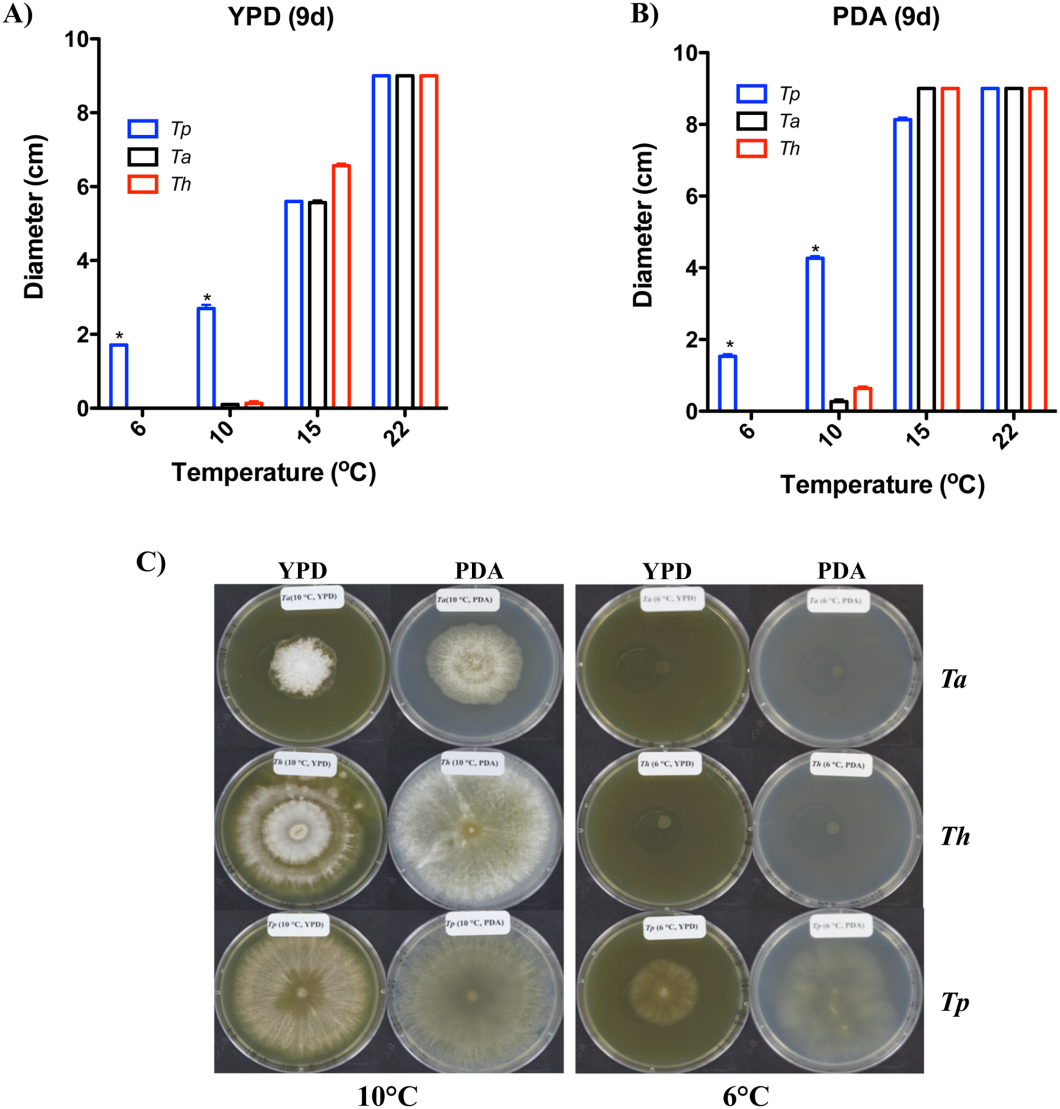
Growth comparisons of *Tp* WPM 39143 at different temperatures. *Tp* WPM 39143 along with *Th* and *Ta* strains were point inoculated on the center of PDA (A) and YPD (B) agar plates and incubated at different temperatures (6°C-22°C) for 9 days and colony diameter was measured. The *Tp* WPM 39143 growth was significantly rapid (asterisk denotes p<0.001) as compared to *Ta* or *Th* at lower temperatures of 6°C and 10°C. Further incubation (20 days) revealed good growth of *Th* and restricted growth of *Ta* at 10°C, but no growth of *Th* or *Ta* at 6°C (C).

In order to examine the biocontrol potential of *Tp* WPM 39143, we first tested its growth at different temperatures in comparison to the well-known biocontrol strains *Th* and *Ta*. Our results indicated that *Tp* WPM 39143 grew fairly well at 6°C while *Th* and *Ta* did not grow at all at 6°C after 9 days post-incubation (Fig 2A and 2B). Even prolonged incubation of 20 days did not support the growth of either *Th* or *Ta* at 6°C while restricted growth of *Ta* was observed at 10°C (Fig 2C). *Tp* 39143 grew better at ≥10°C but growth was also evident at 6°C, a temperature more prevalent in the caves and mines [40].

### Dual culture challenge studies

**Fig 3:**
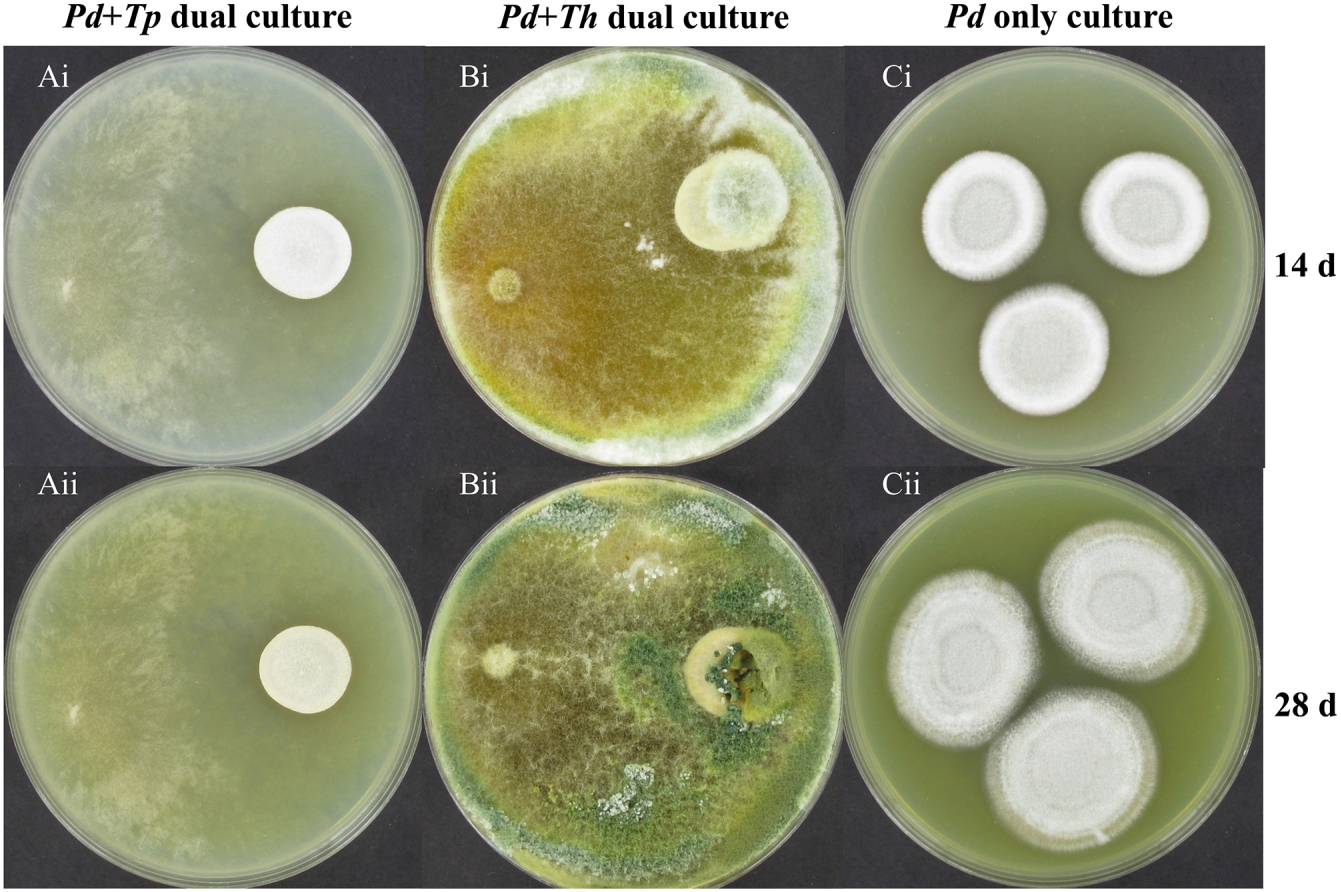
Dual culture challenge on PDA agar medium. Approximately 15 μl spore suspension of *Pd* (10^7^/ml) was inoculated near the edge of the PDA plate. Following incubation at 15°C for 10 days, 15 μl of *Tp* WPM 39143 or *Th* conidial suspension (10^5^ cells/ml) was inoculated on the opposite edge of the culture plate and the interaction between these two fungi was assessed at 14 and 28 days post-incubation. *Pd* colonies were white and restricted in the presence of *Tp* WPM 39143 or *Th* as compared to fluffy and pigmented when grown alone.

In the dual culture challenge experiments on PDA agar, the *Pd* hyphal extension (diameter) was 1.93 ± 0.1 cm in the presence of *Tp* WPM 39143 (Fig 3Ai) while it was 2.82 ± 0.2 cm in the absence of *Tp* WPM 39143 at 14 days post-incubation (Fig 3Ci). No extension of *Pd* hyphal growth was observed in the presence of *Tp* WPM 39143 (Fig 3Aii) while it steadily increased to 3.90 ± 0.2.cm at 28 days post-incubation (Fig 3Cii). Of particular interest, *Pd* colonies in the presence of *Tp* WPM 39143 appeared white and restricted as compared to fluffy and pigmented when grown alone (Fig 3Ci and 3Cii). A similar trend was observed for *Pd* grown in the presence of *Th* (Fig 3Bi and 3Bii).

**Fig 4:**
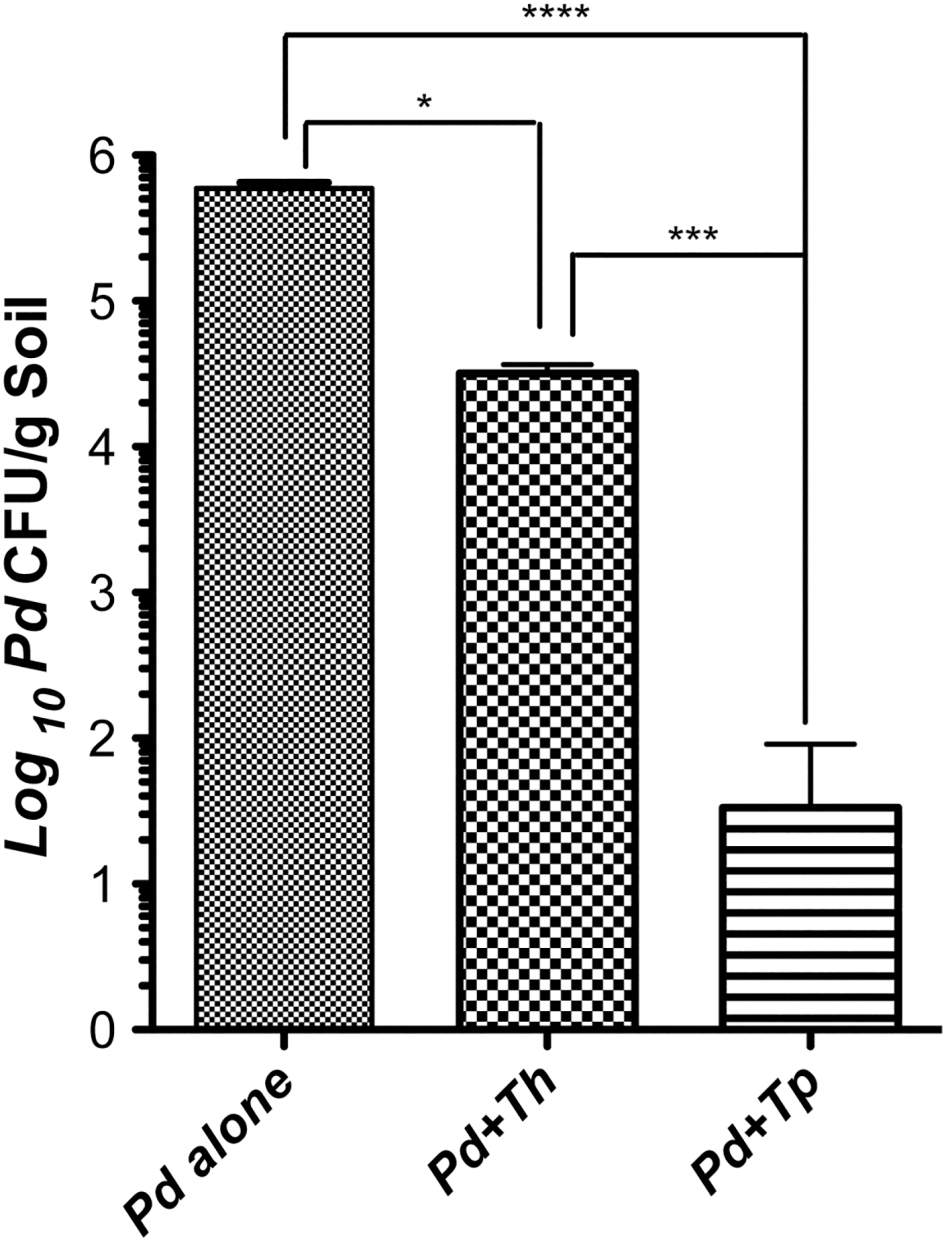
*Pd* CFU recovery from dual culture challenge in soil (culture-dependent method). Autoclaved soil sample was first inoculated with 100 μl of *Pd* conidia (10^4^/g) and following incubation for 7 days at 15°C, one soil sample each containing *Pd*, was inoculated with 100 μl of *Tp* WPM 39143 or *Th* conidia (10^4^/g) or 100 μl of water alone. Dual culture challenge samples and control samples were incubated at 15°C for another 35 days, and three aliquots of 100 mg soil from each treatment group were processed for the recovery of *Pd* in culture. Approximately, 4-logs reduction of *Pd* CFU by *Tp* WPM 39143 (*p*<0.0001) compared to 1.7-logs reduction of *Pd* CFU by *Th* (*p*<0.05) was observed.

**Fig 5:**
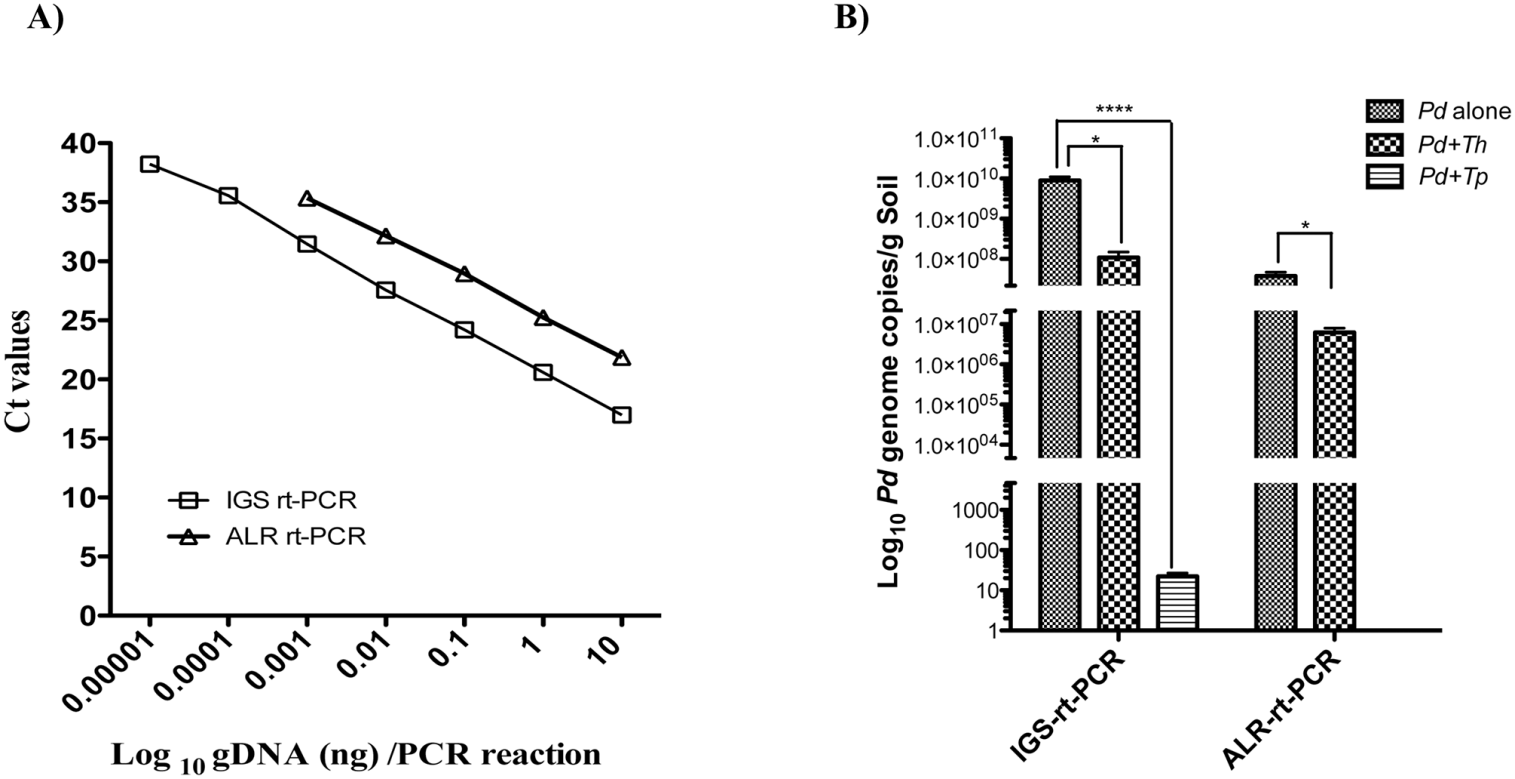
*Pd* genome copies recovery from dual culture challenge in soil (culture-independent method). A, Standard curves of *Pd* intergenic spacer region (IGS) and alpha L-Rhamnosidase (ALR) gene by real-time PCR assays. The purified genomic DNA from *Pd* was used for the generation of standard curve against IGS and ALR targets, which was used to extrapolate *Pd* genome copies in the soil samples with or without biocontrol agents. All data points represent the mean *Ct* value of amplification reactions done in triplicates with error bars denoting standard deviation. The assay was linear over 6 orders of magnitude for IGS gene (*y* = –3.5886*x*+42.131, R^2^ = 0.9982) and over 4 orders of magnitude for ALR gene (*y* = –3.398*x*+45.716, R^2^ = 0.9994). B, *Pd* genome copies recovery by real-time PCR assays. Autoclaved soil sample was first inoculated with 100 μl of *Pd* conidia (10^4^/g) and following incubation for 7 days at 15°C, one each soil sample containing *Pd* was inoculated with 100 μl of *Tp* WPM 39143 or *Th* conidia (10^4^/g) or 100 μl of water alone. The dual challenge samples were incubated at 15°C for additional 35 days and three aliquots of 100 mg each soil sample from each treatment group were processed for *Pd* gDNA extraction followed by IGS and ALR real-time PCR assays. The mean *Ct* counts equivalent to DNA was extrapolated from the standard curve, and *Pd* genome copies were calculated based on the formula described in Materials and Methods. Substantial reduction of *Pd* genome copies observed in samples challenged with *Tp* WPM 39143 (p<0.0001) as compared to samples challenged with *Th* (*p*<0.05).

Next, we determined if *Tp* WPM 39143 induced growth inhibition was sustainable in autoclaved soil sample mimicking the natural environment. This information is crucial if *Tp* WPM 39143 is to be considered as a biocontrol agent of *Pd* in caves and mines. The *Th* induced growth inhibition was also determined for comparison. The autoclaved soil sample supported good growth of *Pd* (10^6^ CFU/g soil) and *Tp* or *Th* (approximately 10^8^ CFU/g soil). When compared to the control soil sample harboring *Pd* alone, the recovery of *Pd* from soil samples harboring *Tp* WPM 39143 was reduced by approximately 4.0-logs (99.98% inhibition). In contrast, the recovery of *Pd* from soil samples harboring *Th* was reduced by 1.7-log (89% inhibition) (Fig 4). A similar trend was observed for *Pd* genome copies in soil samples by real-time PCR assay. As compared to the recovery of *Pd* genome copies from control soil sample harboring *Pd* alone, several logs reduction of *Pd* genome copies was observed in soil samples harboring *Tp* WPM 39143, and only one log reduction of *Pd* genome copies was observed in soil samples harboring *Th* (Fig 5). These results confirmed that *Tp* WPM 39143 had potent inhibitory activity against *Pd*, and the inhibitory activity was sustainable under a simulated natural setting.

### Inhibitory activity of *Tp* WPM 39143 extract

**Fig 6:**
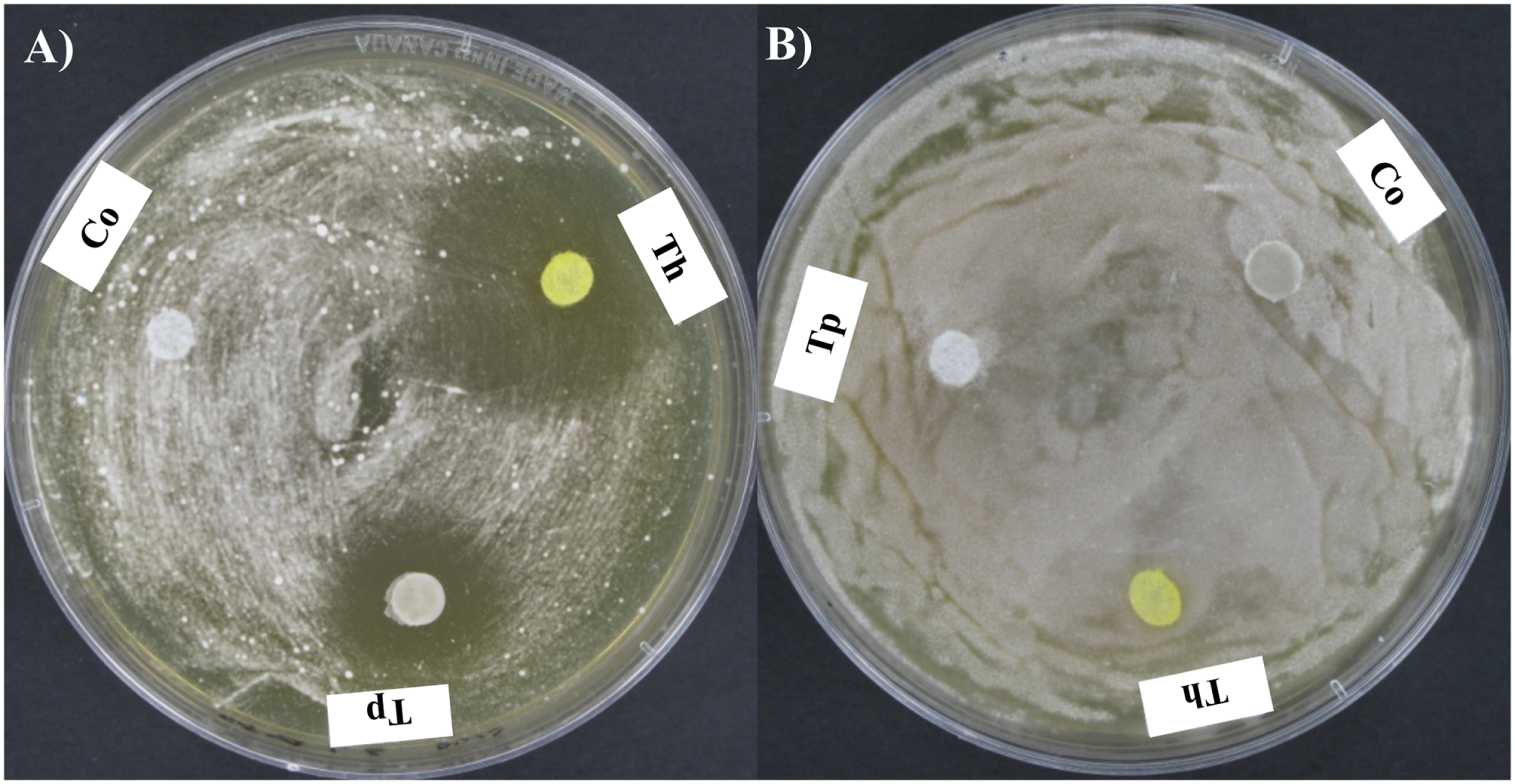
Inhibition of *Pd* by *Tp* WPM 39143 and *Th* extracts. YPD agar plates were streaked with 10^8^ spore suspensions of *Pd* or *Pp*. Sterile filter discs (6 mm) were placed on the surface of the inoculated plate and saturated with 15 μl of *Tp* WPM 39143 or *Th* crude extract. Plates were incubated at 15°C for 10 days. Both *Tp* WPM 39143 and *Th* extract showed inhibitory activity against *Pd* (A) but not against *Pp* (B). The fermentation medium without metabolites, which went through similar extraction process as medium containing metabolites served as a negative control (NCo).

In order to identify active ingredients for biocontrol, preliminary experiments were done with crude extract of *Tp* WPM 39143 against *Pd*. We also included crude extracts from well-known biocontrol fungi *Th* and *Ta* for comparison. *Pseudogymnoascus pannorum* (*Pp*), a species closely related to *Pd* and abundant in caves and mines [20] was also included in the challenge experiment as a target to compare species-specific biocontrol activities. The crude extract from *Tp* WPM 39143 showed inhibitory activity against *Pd* as judged by the presence of clear zone surrounding the disc (Fig 6A) but not against *Pp* (Fig 6B). Similarly, the *Tp* WPM 39143 crude extract was not inhibitory against fungi common in caves and mines including *Aspergillus* species, *Penicillium* species, *Mucor* species, *Fusarium* species, *Cladosporium* species, *Alternaria* species and *Paecilomyces* species (data not shown). The *Th* crude extract also showed more pronounced inhibitory activity against *Pd* but not against *Pp* (Fig 6A and 6B). The *Ta* crude extract did not inhibit either *Pd* or *Pp* (data not shown). These results indicated that the inhibitory metabolites in *Tp* or *Th* crude extracts might be specific against *Pd*.

### Characterization of *Tp* WPM 39143 inhibitory metabolites

**Fig 7:**
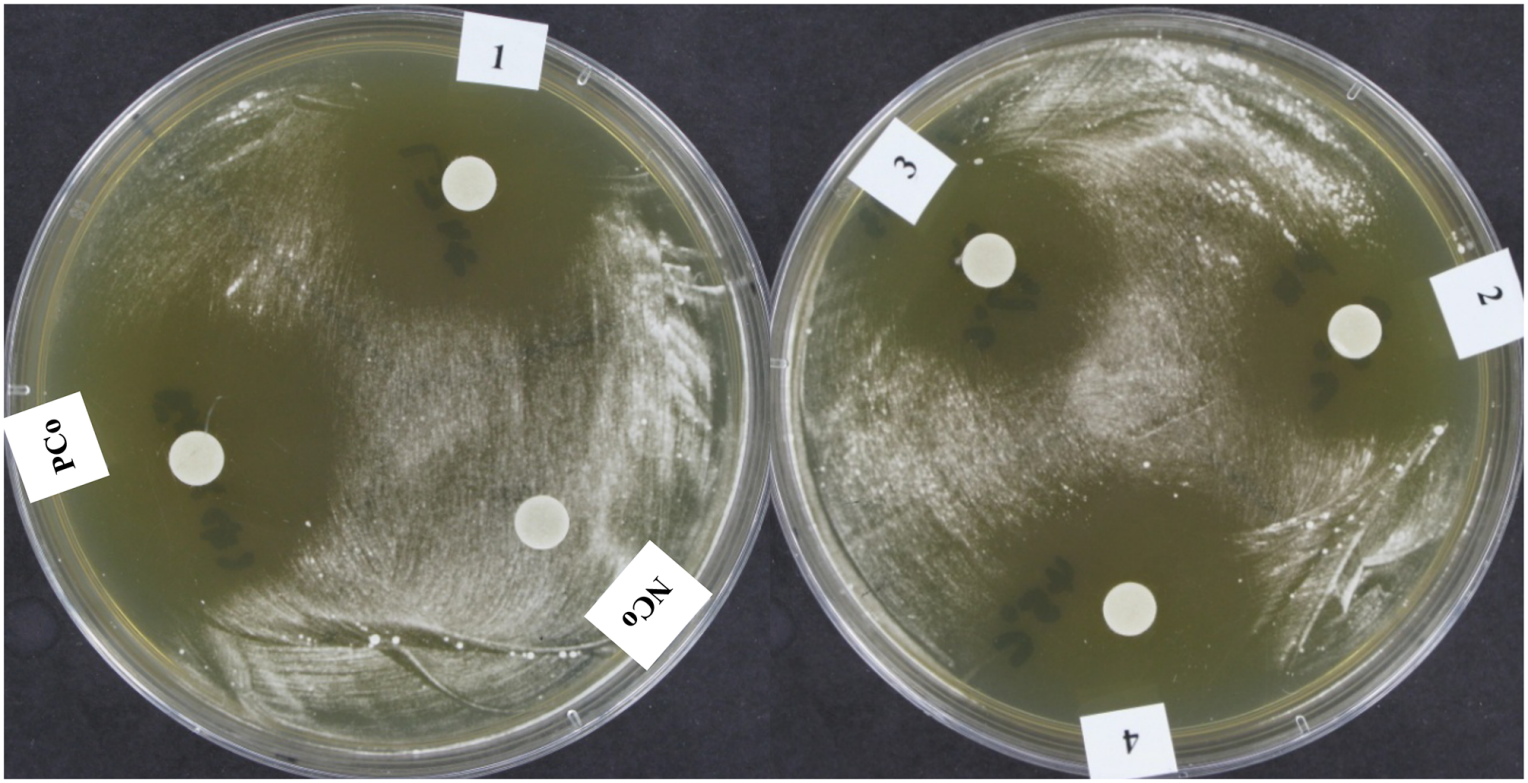
Stability assessment of *Pd* inhibitory activity in *Tp* WPM 39143 crude extract. YPD agar plates were streaked with 100 μl of *Pd* conidial suspension (10^8^) and sterile filter discs (6 mm) were placed on agar plates and saturated with 15 μl of treated or untreated *Tp* WPM 39143 extract. Plates were incubated at 15°C for 10 days and were photographed for inhibition zone around discs. Abbreviations: PCo, positive control (untreated *Tp* WPM 39143 extract), NCo, negative control (fermentation medium alone); 1, *Tp* WPM 39143 extract exposed to regular light for 20 h; 2, *Tp* WPM 39143 extract exposed to proteinase K at 37°C for 20 h; 3, *Tp* WPM 39143 extract exposed to proteinase K at 45°C for 20 h; 4, *Tp* WPM 39143 extract exposed to 45°C for 20 h.

Since *Tp* WPM 39143 grew in the laboratory at cave and mine temperatures (6°C-15°C) and exhibited *Pd* inhibition, we further assessed its inhibitory potential by determining the stability of metabolites at different temperatures, light exposure, and proteinase K treatment. The treated extracts inhibited *Pd* growth as efficiently as non-treated extract indicating that *Tp* WPM 39143 extract is stable at high temperatures and under regular light (Fig 7).

**Fig 8:**
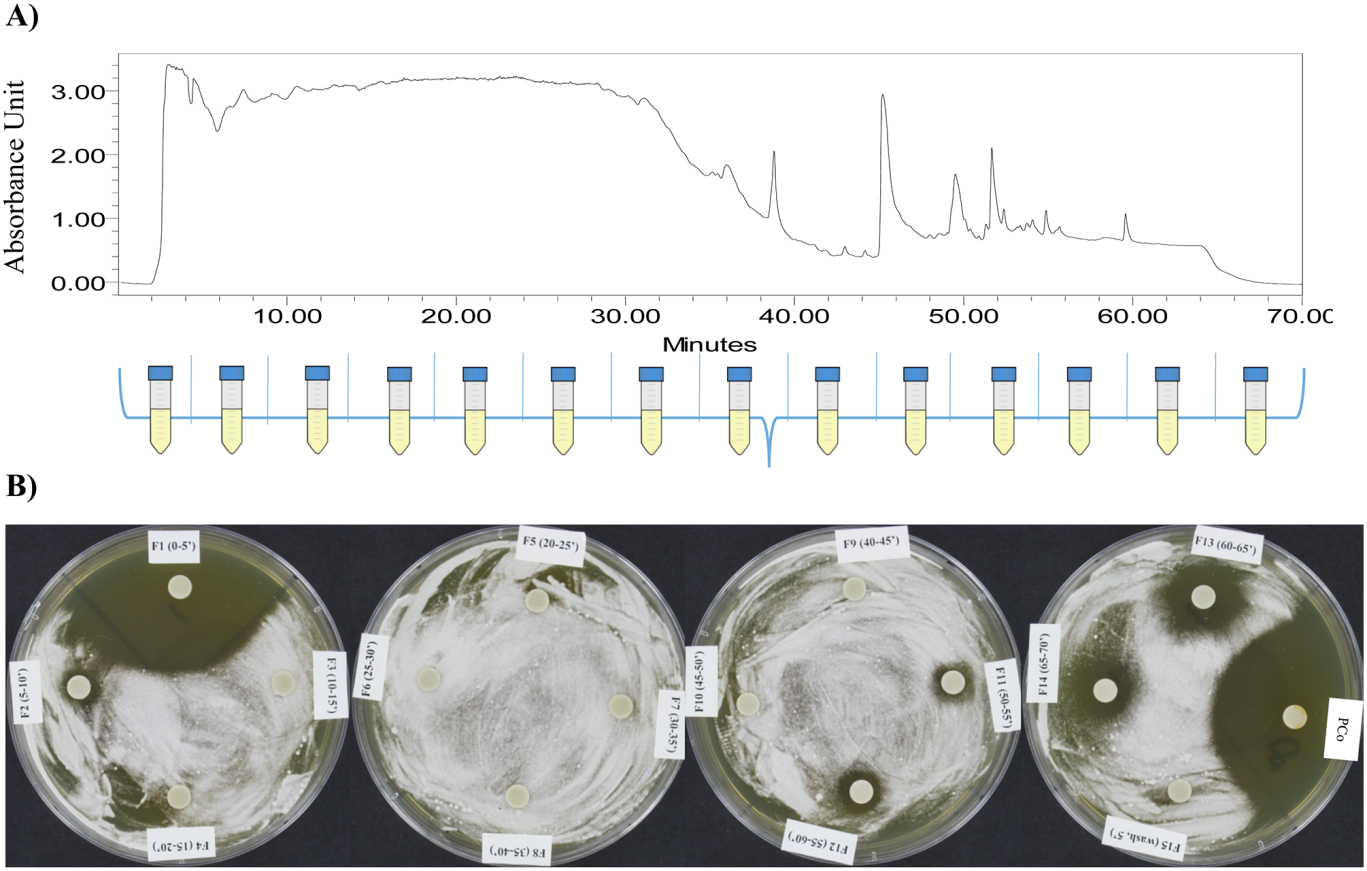
Inhibitory activity of HPLC fractions obtained from *Tp* WPM 39143 extract. A) *Tp* WPM 39143 crude extract was fractionated by HPLC (details in methods). Total of 15 sub-fractions were collected at the intervals of 5 min. B) Disc diffusion assay to measure inhibition of *Pd* growth by various HPLC sub-fractions. F1, 0–5 min sub-fraction; F2, 5–10 min sub-fraction; F3, 10–15 min sub-fraction; …………. F14, 65–70 min sub-fraction; F15, 70–75 min, 5% CH_3_OH in H_2_O elution time. Abbreviation: PCo, positive control (untreated *Tp* WPM 39143 crude extract).

Since *Tp* WPM 39143 extract was not altered by proteinase k treatment indicating that the inhibitory metabolite(s) in *Tp* WPM 39143 extract are presumably not proteins, but a mixture of compounds (Fig 7). Therefore, we explored the chemical nature of inhibitory metabolites in *Tp* WPM 39143 crude extract. The *Tp* WPM 39143 crude extract was fractionated using high performance liquid chromatography (HPLC) and a total of 15 fractions were collected (Fig 8A). The first two sub-fractions (F1, 0–5 min and F2, 5–10 min-red arrow) and the last four sub-fractions (F11, F12, F13, and F14 –yellow arrow) displayed inhibitory activity against *Pd* while other fractions did not (Fig 8B). These results indicated that there are two major subclasses of compounds in *Tp* WPM 39143 extract, high polarity compounds with higher inhibitory activity to *Pd* and low polarity compounds with lower inhibitory activity to *Pd*.

## Discussion

The major findings of this study were: the isolation of novel *Tp* WPM 39143 strain from a WNS affected cave, growth of the novel strain at temperatures simulating cave and mine environments, and potent and specific inhibitory activity of *Tp* WPM 39143 against *Pd* in laboratory media and autoclaved soil. Biocontrol agents are already accepted as an environmentally friendly alternative to chemicals for plant disease management in agriculture [54, 55]. Recently, a zooplankton *Daphnia magna* was suggested as a promising biocontrol agent for *Batrachochytrium dendrobatitis*, a chytrid fungus responsible for deadly chytridiomycosis in amphibians [56, 57]. Our findings of a novel, psychotolerant *Tp* WPM 39143 now sets the stage for potential biological decontamination of *Pd* at WNS affected sites.

There were no published studies prior to a report from our laboratory that documented *Tp* from caves and mines [20]. We isolated *Tp* from only William Preserve Mine, and that too from 3 of the 25 samples investigated. The rare isolation indicated that it is probably not present in large numbers as are some other fungi or has a restricted niche. Since, we did not observe any inhibition of at least some of the major fungal genera by *Tp* apart from *Pd*, it follows that *Tp* could serve as a biocontrol agent for the inhibition or eradication of *Pd* from caves and mines. The excellent growth of *Tp* observed in autoclaved soil sample from Aeolus Cave suggests that the organic and inorganic contents of the soil favor the growth of *Tp*. Additionally, our limited studies documented the fungicidal activity of *Tp* against *Pd* in spiked autoclaved soil sample. These results further confirmed the potent biocontrol activity of *Tp* against *Pd* and the biocontrol activity was sustainable under simulated natural setting. Therefore, the stage is set for future experiments to demonstrate if *Tp* can grow well in the presence of other microbes in natural settings and if it can sustain inhibitory activity against *Pd*.

*Trichoderma* spp. are prominent among the most effective mycoparasites. Several species such as *T*. *harzianum*, *T*. *polysporum*, *T*. *viride* and *T*. *virens* are currently in commercial production for the control of plant pathogenic fungi in agriculture and horticulture [24, 58]. A number of studies have documented that psychrophilic range limits the biocontrol potential of *Trichoderma* spp. and only a few cold adapted strains with biocontrol potential have been reported, including *T*. *aureoviride*, *T*. *harzianum*, and *T*. *viride* [35, 59]. Although *T*. *polysporum* has been reported from cold environments including the Arctic and Antarctic, none of the reported strains are known for their potent biocontrol activity against other fungi [60, 61]. Thus, the discovery and characterization of *Tp* WPM 39143 establishes it as a potentially unique biocontrol agent against the psychrophilic, zoopathogen *Pd*.

*Trichoderma polysporum* secondary metabolites include cyclosporin [62, 63], trichosporin [64], peptaibols [65], anthraquinones [38], trichodermin [24], and minor cyclonerodiol derivatives [66]. Among them, trichosporin, cyclosporine, peptaibols, and cyclonerodiol derivatives have antifungal activities [62, 63, 66]. Many of these metabolites are amphipathic [67, 68]. It is relevant to recall that water soluble, high polarity compounds with specific inhibitory activity against *Pd* were identified in our preliminary analyses. The next logical experiments will be to define the fungicidal molecule (s) in *Tp* extracts active against *Pd*.

In conclusion, we have identified a novel biocontrol strain *Tp* WPM 39143 from a cave at the epicenter of the WNS zoonotic. *Trichoderma polysporum* WPM 39143 produced potent inhibitory compound(s) that impeded the growth of *Pd* in both laboratory media and soil matrices. These results could be used to design a viable strategy for the biological decontamination of *Pd* in ‘cave or mine in a lab’ environment or under field conditions.

## Supporting Information

S1 Fig Flowchart of dual culture challenge in soil. Flow chart representing culture-dependent (CD) and culture-independent (CI) methods for the recovery of *Pd* in the presence or absence of biocontrol fungi, *Tp* WPM 39143 or *Th*.
(TIF)
