# Supplementary figures and images for: Novel Trichoderma polysporum Strain for the Biocontrol of Pseudogymnoascus destructans, the Fungal Etiologic Agent of Bat White Nose Syndrome
Source: PLoS One. 2015 Oct 28;10(10):e0141316. doi: 10.1371/journal.pone.0141316 (PMC4624962; doi:10.1371/journal.pone.0141316)

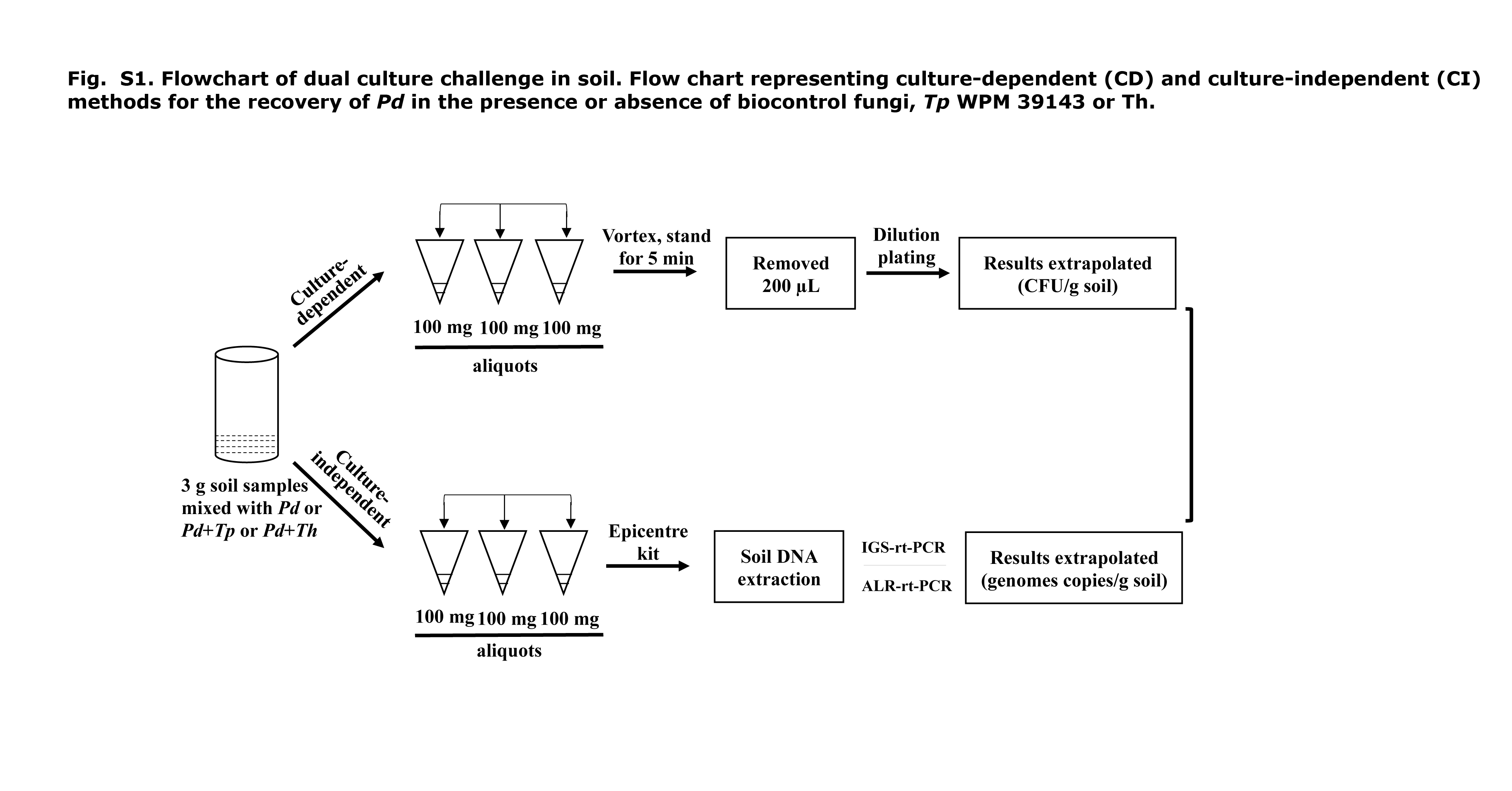

Supplement: S1 Fig — Flow chart representing culture-dependent (CD) and culture-independent (CI) methods for the recovery of Pd in the presence or absence of biocontrol fungi, Tp WPM 39143 or Th. (TIF) [file pone.0141316.s001.tif]
